# Transumbilical laparoendoscopic single-site surgery for pregnancy complicated with ruptured giant ovarian teratoma in the third trimester: case report

**DOI:** 10.3389/fmed.2025.1519205

**Published:** 2025-10-23

**Authors:** Mei-Qin Gong, Yong-Qing Zhang, Ya-Yi Hu, Xiao-Dong Wang

**Affiliations:** ^1^Department of Obstetrics and Gynecology, West China Second University Hospital, Sichuan University, Chengdu, China; ^2^School of Computer Science, Chengdu University of Information Technology, Chengdu, China

**Keywords:** pregnancy with ovarian mass, diagnosis, treatment, pregnancy operation, laparoendoscopic single-site surgery

## Abstract

**Background:**

Most adnexal masses are incidental findings during pregnancy and usually resolve spontaneously. However, pregnancy complicated by a giant ovarian mass is rare, and surgical intervention is recommended when the mass exceeds 10 cm in diameter or persists during the pregnancy. Traditional laparoendoscopic surgery often requires extended periods of pneumoperitoneum, and the need for an initial blind puncture increases the risk of damaging the pregnant uterus and ovarian mass. With advancements in transumbilical laparoendoscopic single-site surgery, operating through an umbilical incision minimizes the potential harm associated with traditional laparoscopy, enhancing the safety of both the mother and fetus. Most literature reports operations conducted during the second trimester when the size and position of the uterus and placenta are optimal, ensuring stable placental function and a low risk of complications such as abortion or preterm birth. As the pregnancy progresses into the third trimester, the uterus moves approximately three transverse fingers above the umbilicus, making it extremely difficult to access a ruptured ovarian mass located posterior to the uterus using single-port laparoscopy. Nevertheless, with continuous improvements in transumbilical laparoendoscopic single-site surgery techniques, as well as the combination of long and short surgical instruments, it is feasible to address the rupture of a giant ovarian mass during the third trimester. Few reports currently detail the use of transumbilical laparoendoscopic single-site surgery for adnexal masses in the third trimester. This report presents a case completed at our hospital.

**Case presentation:**

We report the case of a giant ovarian tumor identified by ultrasound in the first trimester of pregnancy in a 27-year-old woman. Due to signs of threatened abortion, conservative treatment was chosen to allow the pregnancy to continue. The giant ovarian mass ruptured at 28^+2^ weeks of gestation, and it was successfully managed using transumbilical laparoendoscopic single-site surgery. The patient achieved a successful pregnancy, delivering at 38^+5^ weeks via emergency cesarean section due to oligohydramnios. We followed up with the mother and newborn for nearly 12 months, and they were healthy.

**Conclusion:**

Routine abdominal or vaginal ultrasound examinations before pregnancy are essential when a giant ovarian mass is detected in the first trimester. This helps prevent complications such as mass rupture, torsion, and adverse fetal outcomes. If surgical intervention is deemed necessary, the second trimester is generally the most appropriate time for evaluation. By this stage, the size and position of the uterus and placenta are stable, the placental function is sound, uterine sensitivity is lower, and the risk of miscarriage, premature birth, and other complications is reduced. For pregnant women with giant ovarian masses who exhibit signs of abortion in the second trimester and do not opt for surgical treatment, transumbilical laparoendoscopic single-site surgery can be considered the preferred method for addressing a mass rupture in the third trimester.

## Background

Most masses detected in early pregnancy are physiological and resolved. However, continuous enlargement of ovarian masses, giant ovarian masses, suspected malignant masses, acute abdomen, and other acute clinical manifestations during pregnancy require surgical treatment (1, 2). Pregnancy complicated with a giant ovarian mass is rare; surgical intervention is recommended when the diameter of the ovarian mass is greater than 10 cm or persists during pregnancy (3, 4). Presently, the definition of pregnancy with a giant ovarian mass is unclear at home and abroad; according to existing research reports, an ovarian mass with a diameter of 10 cm or more is temporarily defined as a giant ovarian mass (5). The most common pregnancy complicated with giant ovarian mass is benign tumors, among which mature ovarian teratoma is the most common, up to 40% (6).

The surgical methods can be divided into laparotomy and minimally invasive surgery (7, 8). Due to potential complications, including more extensive wounds, prolonged recovery times, and stress reactions that may lead to abortion, laparotomy is no longer the preferred surgical approach during pregnancy. According to the research of domestic and foreign scholars, the most appropriate time for surgery is between 16 and 20 weeks of gestation, when the size and position of the uterus and placenta are applicable, the placental function is stable, the uterine sensitivity is low, and the risk of complications such as abortion and premature birth is low (9).

Although traditional laparoscopic surgery dramatically reduces the trauma, the first blind puncture operation has the risk of damage to the enlarged pregnant uterus or ovarian mass; In recent years, with the gradual maturity of laparoendoscopic single-site surgery, its application in pregnancy adnexal surgery has the advantages of less trauma, quick recovery, quick and safe specimen collection, and little impact on pregnancy, at the same time, as the umbilicus ostomy is an open operation, it could avoid the possible injury caused by the first blind puncture operation (pneumoperitoneum needle or trocar), the safety of mother and fetus is guaranteed. According to the existing literature reports, transumbilical laparoendoscopic single-site surgery is mainly applied to ovarian mass in the second trimester. Still, almost no reports have been reported in the third trimester. Here, we report a case of transumbilical laparoendoscopic single-site surgery applied in the surgical treatment of ruptured ovarian mass in the third trimester of pregnancy; finally, the pregnant woman was successfully pregnant to term delivery, and the follow-up for nearly 12 months showed good maternal and child outcomes.

## Case presentation

A 27-year-old primigravida who had spontaneously conceived presented to our hospital for routine ultrasound examination indicating intrauterine pregnancy. Doppler ultrasound demonstrated a heterogeneous hyperechoic mass measuring approximately 9.7 × 7.0 × 9.6 cm in the right adnexal region, highly suspicious for an ovarian teratoma. tumor marker examination also showed an increase in AFP of 302 ng/ml. At 12^+3^ weeks, ultrasound scanning showed a nuchal translucency thickness of 2.4 mm, the ovarian mass was further enlarged to approximately 11.2 × 6.8 × 6.0 cm. The gynecologist informed the pregnant woman of the risks associated with conservative treatment or surgical treatment, however, the pregnant woman insisted on because of the presence of fetal membrane dissection of about 3.7 × 2.4 cm. Our regular Doppler ultrasound reexaminations indicated that fetal membrane detachment was indeed persistent, with the maximum size being 5.4 × 1.6 × 4.4 cm. She was admitted to our hospital due to left lower abdominal pain for 5 h at 28^+2^ weeks. Five hours before admission, the pregnant woman had persistent pain in the left lower abdomen without obvious inducement, accompanied by a sense of anal distension, no vaginal bleeding, vaginal discharge, nausea, vomiting, or other discomfort. Physical examination revealed tenderness in the lower abdomen and low uterine tone, at the same time, irregular contractions accompanied the pregnant woman.

**FIGURE 1:**
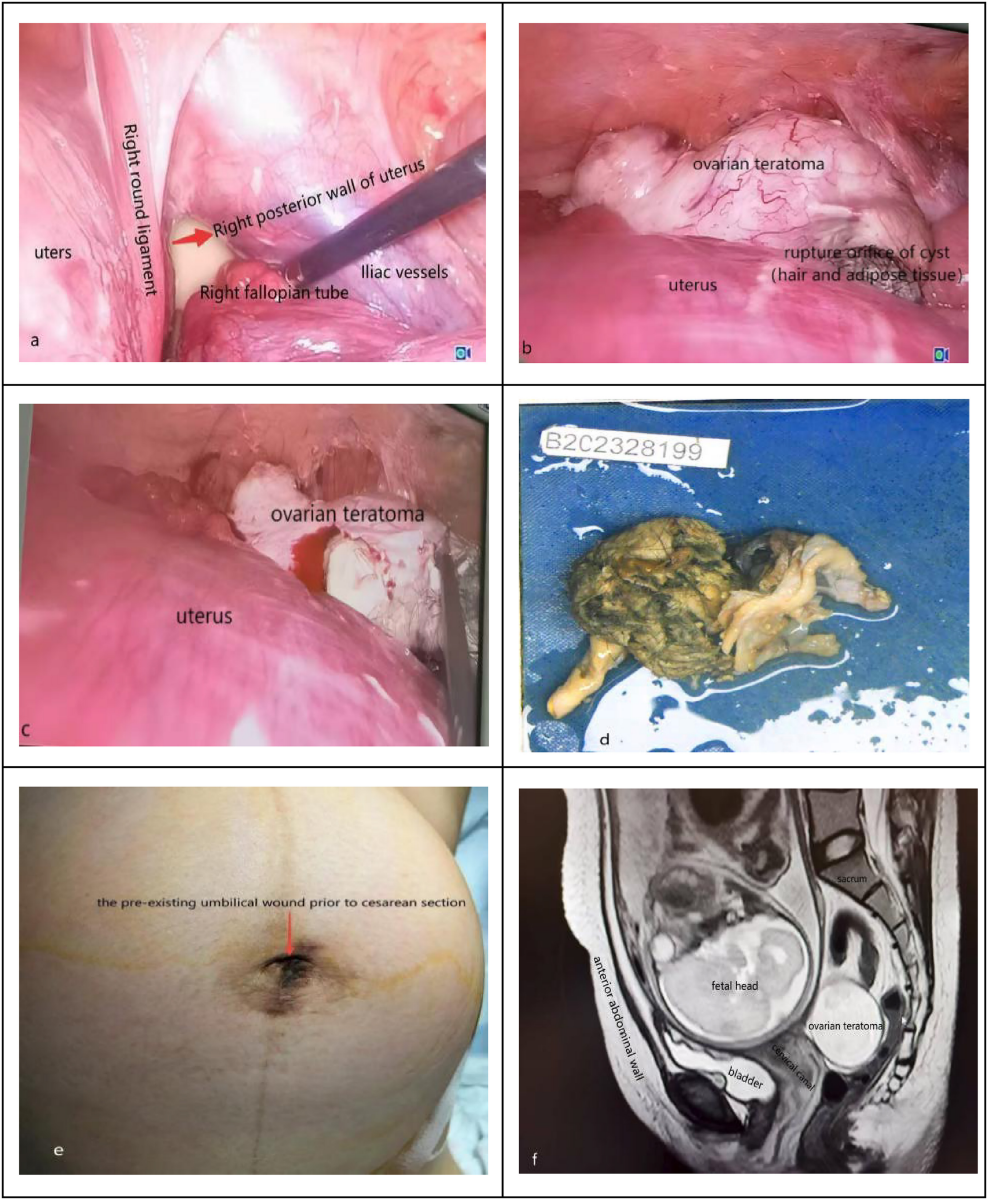
**(a)** Transumbilical laparoendoscopic single-site exploration, the cyst is located in the right posterior part of the uterus; **(b)** the ovarian teratoma was exposed by adjusting the position of the pregnant woman and using instruments, hair and adipose tissue can be seen at the orifice; **(c)** transumbilical laparoendoscopic single-site removal of ovarian teratoma; **(d)** the specimen of the teratoma after stripping; **(e)** the pre-existing umbilical wound prior to cesarean section; **(f)** preoperative MRI suggested that the ovarian teratoma was located posterior and to the right of the uterus.

After admission, treatment with uterine contraction suppression was immediately initiated, and a re-examination of ultrasound showed that there was a cystic-solid mass of about 12.5 × 6.0 × 9.0 cm in size in the right posterior of the uterus, and ovarian teratoma was suspected. We considered that torsion or rupture of the ovarian mass could not be ruled out. The location of the pain that the pregnant woman complained of was inconsistent with the location of the mass indicated by the ultrasound examination. Further pelvic MRI was performed, and a ruptured right ovarian teratoma was highly suspected (Figure 1f). The patient and her family were fully informed of the relevant conditions; transumbilical laparoendoscopic single-site surgery was performed on the second day of admission after obtaining informed consent.

The patient was placed in Trendelenburg position by 15 to 25 degrees during the surgery, tracheal intubated under general anesthesia, and the skin was cut about 2 cm longitudinally in the middle of the umbilicus, then the subcutaneous tissue, fascia and peritoneum were incised in turn, make a port in the umbilicus, pneumoperitoneum was established with a pressure of 12 mmHg (1 mmHg = 0.133 kPa). The lens and operating instruments were inserted through the port of operation for a comprehensive exploration. Initially, we saw a lot of adipose tissue at right posterior to the uterus (Figure 1a). The right ovarian mass was located posterior to the uterus, the ovarian mass was exposed by adjusting the position of the pregnant woman and using instruments, and we found the ovarian cyst with an orifice in which hair and adipose tissue were visible, the appearance of the right fallopian tube was normal (Figure 1b). Next, the procedure of ovarian mass resection was the same as that of traditional laparoscopic surgery.

Due to the “chopstick effect” (10) in single-port laparoscopic surgery, we used long instruments and short instruments to cooperate in the operation to reduce the difficulty of surgery (Figure 1c). The cyst wall tissue and contents were completely stripped out and sent for pathological examination (Figure 1d), the ovarian cortex was sutured with absorbable sutures. The mass was put into the specimen bag and removed through the umbilical incision. After the operation, the pelvic and abdominal cavities were rinsed, and then pull out the lens and instruments. The peritoneum and fascia of the umbilicus incision were sutured with non-absorbable thread, and the umbilicus plasty was performed by “anchor suturing technique” (11). The intraoperative blood loss was about 100 ml, and the procedure was approximately 2 h long. The fetal heart rate and uterine contraction were monitored after the operation. Antibiotics were used to prevent infection for 24 h, and atosiban was used to inhibit uterine contraction for 48 h. The final pathologic diagnosis was maturity ovary teratoma. The patient recovered well; fetal heart rate and fetal movement were normal, obstetric ultrasound and blood routine examination showed no abnormality, without abdominal distension, abdominal pain, vaginal bleeding, and discharge fluid, and then discharged from the hospital on the third day after the operation. The pregnant woman continued regular prenatal examinations until 38^+5^ weeks of gestation, the pre-existing umbilical wound prior to cesarean section as (Figure 1e). Emergency cesarean section was performed because of oligohydramnios, and both mother and child were in good condition.

## Discussion and conclusions

Adnexal mass is a common disease in obstetrics and gynecology, and the majority of cysts detected in early pregnancy are physiological and resolved; However, acute clinical manifestations such as continuous enlargement of ovarian masses during pregnancy, giant ovarian masses, suspected malignant tumors and acute abdomen require surgical treatment (12). Laparoscopic surgery has become the preferred treatment for many non-obstetric abdominal surgeries in pregnant women. According to the 2020 British Society of Gynecologic Endoscopy evidence-based guidelines on laparoscopy in pregnancy, laparoscopic minimally invasive surgery for pregnancy with ovarian masses does not increase the risk of miscarriage, premature delivery, and fetal malformations (13). There are many reports on the application of transumbilical single-port laparoscopy in the second trimester of pregnancy to complete adnexal surgery in recent years. In 2013, Polat Dursun et al. reported two cases of adnexectomy performed by transumbilical laparoendoscopic single-port laparoscopy during pregnancy. One of the cases was a left ovarian cyst of 18 cm at 12 weeks of pregnancy; Another case was a twin pregnancy patient conceived through assisted reproductive technology, who had torsion of the left adnexal cyst pedicle at 25 weeks, neither of the two patients had fetal or maternal complications during pregnancy after the operation (14). Compared with laparotomy surgery, pregnant women who underwent laparoscopic surgery recovered more quickly and spent a short time in the hospital, with a lower wound infection rate.

According to the research of domestic and foreign scholars, the most appropriate time for surgery is between 16 and 20 weeks of gestation, when the size and position of the uterus and placenta are applicable, the placental function is stable, the uterine sensitivity is low, and the risk of complications such as abortion and premature birth is low (9). In this case, although a giant ovarian cyst was found in the early pregnancy, surgery was not performed because of persistent signs of threatened abortion; We entirely communicated with the patient about the risk of rupture and torsion of the giant cyst. Unfortunately, the giant ovarian cyst ruptured at 28^+2^ weeks gestation. In fact, in terms of technology, transumbilical laparoendoscopic single-site surgery of non-pregnant giant ovarian benign tumors has been relatively mature (15). At present, transumbilical laparoendoscopic single-site adnexal surgery during pregnancy is more often performed in the second trimester, and transumbilical laparoendoscopic single-site adnexal surgery in late pregnancy is rarely reported. The most challenging problem of laparoendoscopic single-site surgery in the third trimester is that the enlarged uterus affects the operation field and space; Secondly, all the instruments are entered into the abdominal cavity by the same incision, and there is mutual interference between the instruments, which requires high technical requirements for the operator (16). Therefore, the difficulty of single-port laparoscopic surgery for benign adnexal tumors in the second trimester is significantly reduced. In this case, surgical treatment was not performed in the second trimester, and the tumor ruptured in the third trimester. Because of the advantages of single-port laparoscopy, laparoendoscopic single-site surgery is still a preferred treatment option. It has been reported in the literature that single-port laparoscopic surgery can effectively reduce the amount of intraoperative bleeding during pregnancy surgery and postoperative pain analog scale (17). At the same time, laparoendoscopic single-site surgery can further reduce incisions and accelerate patients’ postoperative recovery (18–20).

Our experience also shows that transumbilical laparoendoscopic single-site surgery is feasible for the treatment of ovarian mass rupture in the third trimester of pregnancy. Due to the particularity of pregnant patients, pregnancy outcome is an essential indicator for postoperative efficacy evaluation of pregnancy complicated with adnexal masses. Related studies have pointed out that transumbilical laparoendoscopic single-site surgery in pregnant patients will increase the incidence of umbilical hernia, which may be related to the increase in incision tension (13). However, some studies have found that transumbilical laparoendoscopic single-site surgery for pregnancy complicated with gynecological benign tumors does not cause the risk of umbilical hernia in patients (21). The correct suture method for an umbilical incision is critical to reduce the incidence of umbilical hernia. The patient, in this case, underwent umbilical suture surgery by “anchoring method” proposed by Professor Zheng Ying of China; the peritoneum and fascia of the umbilical incision were sutured with non-absorbable suture, and the umbilical plastic surgery with delayed absorption suture (22), the pregnancy was successfully carried to term without umbilical hernia. Intra-abdominal pressure of <15 mmHg during laparoscopy will prevent side effects of pneumoperitoneum (23). Although cardiorespiratory circulation can be affected because of CO_2_ pneumoperitoneum and the Trendelenburg position, as well as fetal acidosis following maternal conversion of CO_2_ to carbonic acid (H_2_CO_3_), laparoscopy can be safely performed in each trimester of pregnancy. Measurement of end-tidal carbon dioxide partial pressure (capnography) in pregnant women can be used for intraoperative CO_2_ monitoring. Respiratory acidosis can be avoided by keeping the end-tidal CO_2_ at 32–34 mmHg (24). Hunter also showed that long-term operation under high-pressure pneumoperitoneum would increase the risk of abortion, fetal acidosis, and fetal hypoxia, among which the changes were most evident under the pressure environment of 15 mmHg (1 mmHg = 0.133 kPa) or above (25). In this case, the pneumoperitoneum was established with a pressure of 12 mmHg, and the newborn was in good condition after nearly 1 year of follow-up.

Combined with our experience, transumbilical single-port laparoscopic treatment of pregnancy with ovarian giant mass is safe, feasible, and has broad application prospects. However, there is no large sample data analysis domestically and abroad, which still needs to be confirmed by further studies in the future. Of note, adnexal tumors with a high degree of suspicion for malignancy are not suitable candidates for laparoendoscopic single-site surgery; patients should be adequately evaluated before surgery.

## Data Availability

The authors acknowledge that the data presented in this study must be deposited and made publicly available in an acceptable repository, prior to publication. Frontiers cannot accept a manuscript that does not adhere to our open data policies.
